# Digital health innovation and artificial intelligence in cardiovascular care: a case-based review

**DOI:** 10.1038/s44325-024-00020-y

**Published:** 2024-10-17

**Authors:** Jelani K. Grant, Aamir Javaid, Richard T. Carrick, Margaret Koester, Ali Asghar Kassamali, Chang H. Kim, Nino Isakadze, Katherine C. Wu, Michael J. Blaha, Seamus P. Whelton, Armin Arbab-Zadeh, Carl Orringer, Roger S. Blumenthal, Seth S. Martin, Francoise A. Marvel

**Affiliations:** 1 Johns Hopkins Ciccarone Center for the Prevention of Cardiovascular Disease, Baltimore, MD USA; 2 Department of Medicine, Division of Cardiology, Johns Hopkins Hospital, Baltimore, MD USA; 3 Department of Internal Medicine, Massachusetts General Hospital, Boston, MA USA; 4 Naples Comprehensive Health System Rooney Heart Institute, Naples, FL USA; 5 Welch Center for Prevention, Epidemiology & Clinical Research, Johns Hopkins University, Baltimore, MD USA

**Keywords:** Cardiac device therapy, Medical imaging, Therapeutics

## Abstract

This narrative review aims to equip clinicians with an understanding of how digital health innovations and artificial intelligence can be applied to clinical care pathways for cardiovascular prevention. We describe a case that highlights augmentative AI for the incidental detection of coronary artery calcium, a mobile application to improve patient adherence/engagement, large language models to enhance longitudinal patient communication and care, and limitations and strategies for the successful adoption of these technologies.

## Introduction

**Fig. 1:**
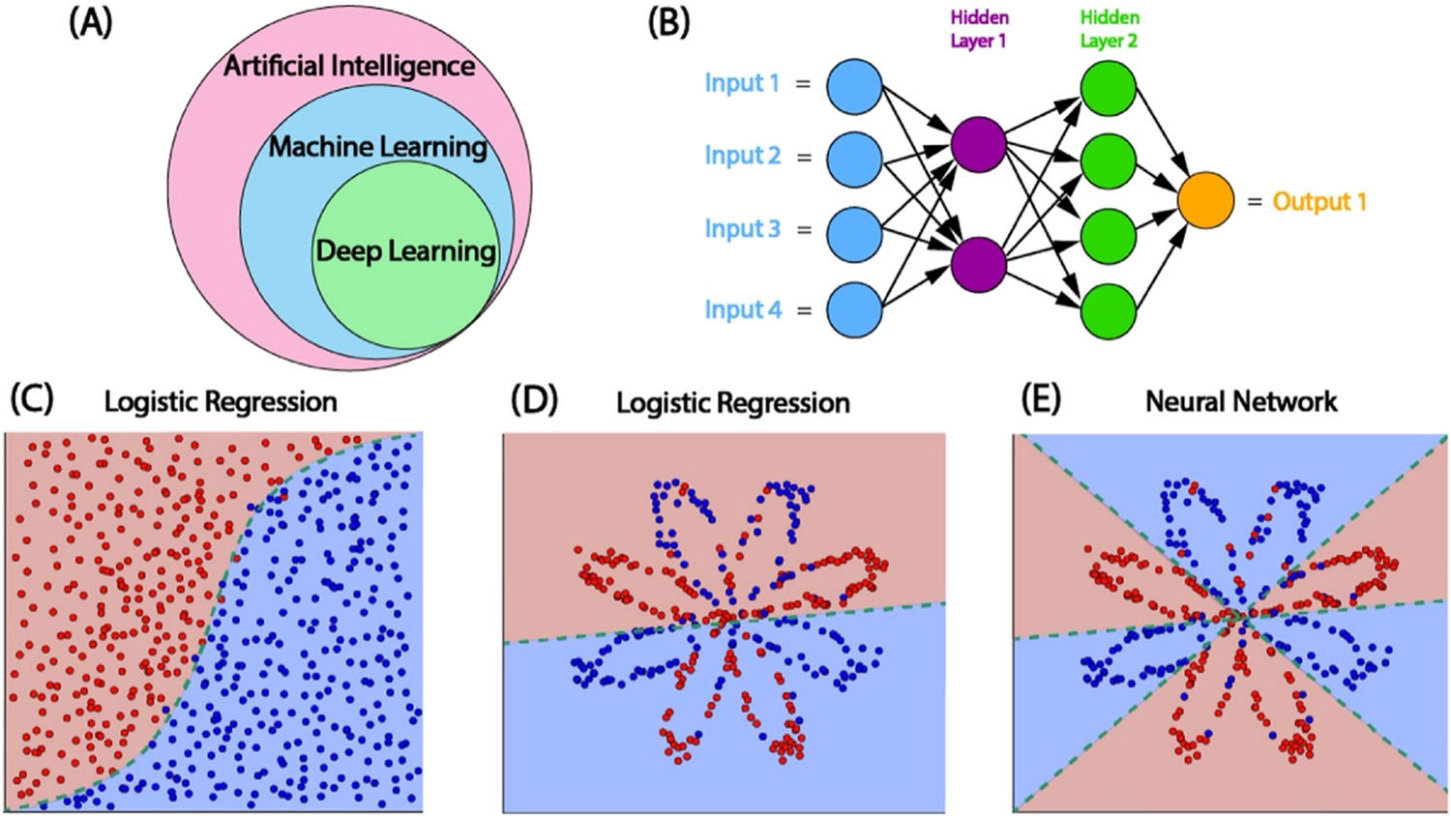
Artificial intelligence overview. **A** Deep learning is a subset of machine learning (ML), which is a subset of artificial intelligence. **B** Architecture of an ML convolutional neural network (CNN) with two hidden layers. >3 layers qualifies as deep learning. **C**–**E** Logistic regression vs. CNN. Traditional cardiovascular risk estimates use logistic regression models, which excel when data is linearly separable (**C**) but not as well in complex situations (**D**). ML can generate more complex decision boundaries (**E**).

The World Health Organization (WHO) has encouraged healthcare systems to prioritize the development, evaluation, implementation, and expansion of digital health innovations (DHI) and to integrate these new technologies into existing health system infrastructures^1^. Similarly, in 2022, the Food and Drug Administration (FDA) commissioned a document focused on advancing the digital health landscape and highlighted the potential of DHI to improve access to care in underserved populations^2^. This has particular relevance for cardiovascular disease (CVD), which remains the leading cause of death worldwide^3^. A focus on lifestyle modification and adherence to effective preventive therapies is the cornerstone of CVD prevention and management, which can be augmented with advancing technology^3^. DHI refers to healthcare delivered via the internet, wearable devices, mobile applications and emerging computational methods leveraging big data and artificial intelligence. Artificial intelligence (AI) is defined as the capability of a machine to imitate intelligent human behavior or perform tasks that normally require human intelligence (Fig. 1). A continuum of AI exists that ranges from situations where machines repeat many human tasks (assisted), enable humans to do more than they are capable of doing (augmented) and fully accomplish tasks on their own without human intervention (autonomous)^1^. The use of AI to improve medical diagnosis and risk assessment has increased dramatically over the past decade^4,5^. Since the FDA first began reviewing AI-enabled devices in 1995, over 800 clinical AI-assisted algorithms have been approved, with cardiovascular disease among the top specialities for FDA-approved AI algorithms^6^.

Technological advancement, including DHI and, in particular, AI, may potentially allow clinicians to tailor treatments specifically to the needs of patients, enabling precision medicine and achievement of improved cardiovascular health^4^. Given the pace of such technological advancements and the subsequent impact on clinical practice, this narrative review aims to equip clinicians with a basic understanding and application of these technologies. We describe a case that highlights (1) augmentative AI for incidental detection of coronary artery calcium (CAC) on chest computed tomography (CT), (2) clinical decision support systems (CDSS) to support adherence to guideline-directed medical therapy, (3) a mobile application to improve patient adherence and engagement, (4) large language models to enhance health coaching for patients, and (5) strategies for successful adoption and governance of these technologies.

**Case Vignette Part 1:** Mr. Anthony Imagine (A.I.) is a 58-year-old man with long-standing hypertension and tobacco use disorder who presents to a primary care practice after a recent emergency department visit for shortness of breath. In the Emergency Department, a non-electrocardiogram (ECG) gated, non-contrast chest CT scan was obtained, which showed a right lower lobe pneumonia, for which he was discharged with a course of antibiotics. He now feels back to his baseline. He has never received routine preventive care. Is there a role for AI to screen for cardiovascular disease based on the testing he received in the Emergency Department?

## Artificial intelligence for the primary prevention of atherosclerotic cardiovascular disease

**Fig. 2:**
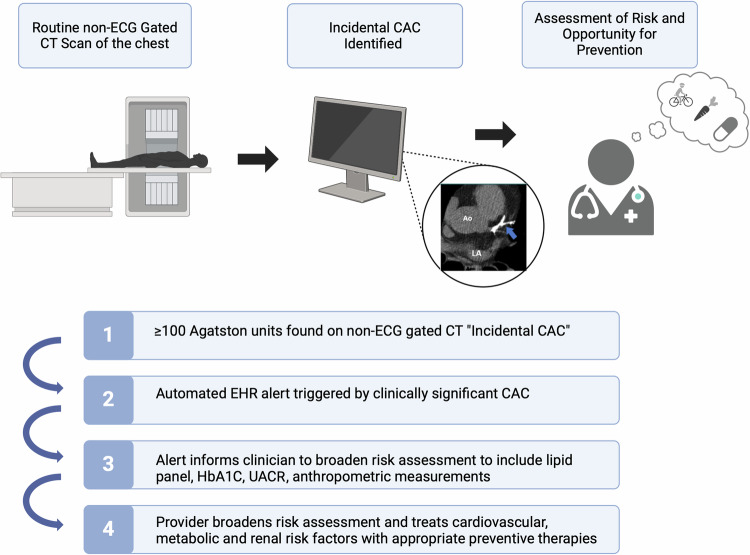
Automatic Identification of incidental CAC by artificial intelligence on a routine, non-ECG-gated chest CT, and the use of clinical decision support systems (CDSS). EHR electronic health record, CAC coronary artery calcium, HbA1C hemoglobin A1C, UACR urine albumin-creatinine ratio.

Mr. A.I. received a non-contrast chest CT scan as part of his workup for shortness of breath. Screening for CAC on his non-gated CT scan of the chest, referred to as “incidental CAC”, provides an opportunity for early identification of increased atherosclerotic cardiovascular disease (ASCVD) risk (Fig. 2). The automatic detection of incidental CAC is achieved using deep learning models to quantify vessel-specific CAC scores on non-ECG-gated chest CTs^5^. Some AI-CAC models use a two-stage process for CAC scoring^6^, such as an atlas for anatomic registration^7^ or a bounding box to define coronary anatomy and discern between coronary and non-coronary calcification (e.g., valvular calcification)^8^. However, the most used algorithm to date uses a single convolutional neural network (CNN) for an end-to end approach^5^. This algorithm was validated internally at Stanford Hospital and externally at four geographically diverse sites within the United States (US) and Latin America. The diagnostic performance for detecting any CAC (≥1 Agatston unit [AU]) was high at all sites (sensitivity range: 82–94% and positive predictive value [PPV] range 87–100%). The specificity and PPV for detecting CAC ≥100 were also high at all sites (specificity range: 93–100% and PPV range 86–93%).

CAC testing is superior to available serum biomarkers and other imaging modalities for atherosclerotic cardiovascular disease (ASCVD) risk assessment^9^. CAC scoring studies, as used in clinical practice, employ ECG-gated imaging (acquisition during 60-80% of the RR interval) to minimize over- and underestimation of calcium. However, recent studies demonstrate that on non-ECG-gated chest CT scans performed for non-cardiac indications, the finding of CAC is similarly predictive of future coronary heart disease risk^5^. Several cardiology and radiology guidelines endorse the routine reporting of CAC on non-gated scans^10–12^. Moderate to severe incidental CAC correlates with an Agatston score ≥100 on a dedicated CAC scan, which is a guideline-based indication for patient-clinician discussion on statin initiation^10^. Despite these recommendations, incidental CAC is underreported by radiologists^13,14^. The main reasons for underreporting are (1) a lack of awareness of guideline recommendations for reporting incidental CAC, (2) a belief that incidental CAC does not significantly impact clinical decision-making, and (3) a lack of radiologist confidence to accurately grade incidental CAC^13,14^. To overcome the underreporting by radiologists, automated detection and reporting of incidental CAC provides a technology-supported approach to ensure universal reporting^14^. As non-contrast chest CT imaging is done far more often than dedicated coronary calcium scoring, CAC reporting on such studies offers the opportunity to provide ASCVD risk information in an expanded number of appropriately selected patients^11^. The NOTIFY-1 project^10^ further demonstrated that opportunistic screening of incidental CAC followed by clinician and patient notification led to a significant increase in appropriately allocated statin prescriptions. Primary care physicians and patients have expressed concern regarding the communication of incidental coronary calcification. The greatest perceived impediments to more widespread reporting of incidental CAC scores and notification include the downstream impact of clinician follow-up burden, lack of clinician understanding, patient anxiety, and inappropriate downstream testing.

The use of AI for risk prediction has expanded beyond the assessment of coronary calcium, with several studies now showing that chest x-rays (CXRs) can be used to detect coronary atherosclerosis^15^ aortic stenosis^16^ and assess left ventricular systolic diameter^17,18^. A recent study^15^ of 8869 outpatients with unknown ASCVD risk sought to develop and test an AI model to estimate 10-year risk for major adverse cardiovascular events (MACE) from routine CXRs and compared its performance to that of traditional ASCVD risk scores to determine statin eligibility. The authors found that those with a ≥7.5% risk as predicted by the CXR-AI algorithm, had a higher 10-year risk for MACE (adjusted hazard ratio [HR], 1.73 [95% CI, 1.47 to 2.03]). In 2132 outpatients with known ASCVD risk, the CXR-AI algorithm predicted MACE beyond the traditional ASCVD risk score (adjusted HR, 1.88 [CI, 1.24 to 2.85])^15^. ASCVD risk prediction continues to evolve, and the expectation is that in the near future, AI can be applied to multiple imaging modalities to further refine risk estimation. However, caution is required to ensure that the imaging input is directly associated with the explored outcome (in this case, ASCVD) to avoid a confounding diagnostic signal based on spurious correlations.

**Case Vignette Part 2:** The results of the AI incidental CAC report quantified a score of 196. How can healthcare systems set up a system to notify appropriate parties of the need for further risk assessment and suggestions for preventive therapies based on this finding?

## Clinical decision support systems

In managing patients like Mr. A.I. who have incidental but clinically actionable results found on CT chest imaging (i.e., moderate to severe or an incidental CAC score ≥100), there is a question of how this information will be shared and who will have ownership in the next clinical management steps. There is no consensus on the best approach to communicating future ASCVD risk after the identification of incidental CAC to the ordering clinician or care team. One strategy that led to greater uptake in statin prescriptions was notifying clinicians of CAC results by sending pictures of the coronary calcium score as demonstrated by NOTIFY-1 project^10^. Implementing computerized CDSS^19^ may improve adherence to clinical guidelines, patient safety, and support cost containment. However, a disadvantage of this system is increased clinician burden, time, and lack of clarity on the actionable next steps in management. Research is ongoing for the best method of communicating risk associated with incidental CAC. A potential approach of using nurse navigators to lead a cardiometabolic clinic or referral to a preventive cardiologist have been proposed^20^.

CDSS are intended to improve healthcare delivery by enhancing medical decisions with targeted clinical knowledge, patient information, and other health information^19^. Usually, CDSS are comprised of a software alert that informs the clinical decision-making process using an input of patient characteristics to derive an evidence-based recommendation. CDSS also includes alert notification systems called Best Practice Advisory (BPAs) which can be “interruptive,” requiring active clinician acknowledgement to proceed or passive “in-line non-interruptive” alerts^21^. The implementation of BPAs is linked to improved workflows, including statin initiation^22^, which was increased with EHR-based clinician alerts.

One advantage of CDSS is the potential increase in adherence to clinical preventive guidelines, follow-up, and treatment. To combat the potential of “alert fatigue,” where too many relatively insignificant alerts or recommendations lead clinicians to dismiss them regardless of importance^21^, critical alerts can be prioritized with a tiered alert system that minimizes the use of disruptive alerts for non-critical indications^21^. To prevent an overreliance on CDSS and ensure clinician autonomy when assisting with clinical management, system designs should not be too “prescreptive” or definitive to ensure an ongoing evaluation of alert impact^19^.

Support tools administered directly to patients through personal health records may help provide patient-focused care. When connected to EHRs, personal health records (PHRs) can promote a two-way relationship, whereby information entered directly by the patient can be available to their clinicians, and information in the EHR can be transmitted to the PHR for patients to view^23^. While the success of such an approach is dependent on the computer literacy of patients, it supports shared decision-making and allows patients to participate in their own care.

**Case Vignette Part 3:** His primary care physician is uncertain about the next steps to manage his overall cardiovascular risk and refers him to a Preventive Cardiology/Cardiometabolic clinic that is managed by a Cardiometabolic coordinator. Is there a role for AI to improve efficiency in such a clinic?

## Large language models

Large language models (LLMs) are AI models that are trained on large amounts of text to recognize, summarize, translate, predict, and generate content^24^. In cardiology, these models provide multiple opportunities to aid patients, clinicians, and researchers^25^. While LLMs in medicine are still in a developmental stage, soon AI and LLMs may serve both to enhance traditional care visits and serve as the bridge between traditional visits and medically supervised home care.

**Fig. 3:**
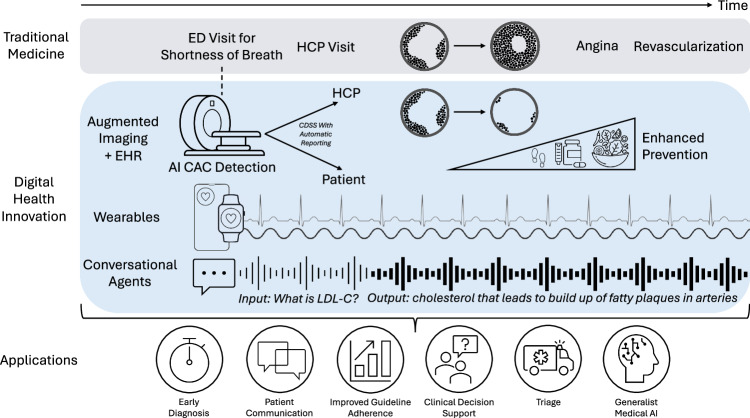
Central figure. Digital health innovation (DHI) and artificial intelligence (AI) in cardiovascular care.

In the near future, LLMs may help to interpret and communicate insights from wearables and home sensors (such as early adverse event detection)^26^, make basic triage decisions^27^, and coordinate follow-up care (Fig. 3)^28^. LLMs are effective at drafting responses to patient questions, which have been rated by patients as being of high quality and more empathetic than physician responses^29^. In the case of Mr. A.I., a clinician could prescribe an AI-assisted coach to serve as a motivator for lifestyle changes, give feedback on dietary habits and physical activity, encourage medication adherence, and gather information that can be used to enhance the efficiency of the next clinical visit. Within a Cardiometabolic Clinic visit, medically trained LLMs may be able to take on the burden of repetitive tasks such as documentation to allow clinicians to dedicate more time to patient care. For example, LLMs may be able to perform chart reviews and summarize salient points before a new patient encounter in a pre-populated note^30^. They may also augment core medical knowledge by creating a template problem list, differential diagnoses, and plan based on the referral indication^31^. During the visit, speech-recognition LLMs can assist with documentation by serving as real-time scribes, differentiating between the voices of participants within the encounter^31^. In cases in which language translation is needed, these models can also serve as interpreters, without the need for a human interpreter. They can also summarize a clinical note and simplify medical language into an after-visit summary with patient instructions, tailored to a specified education level^32^.

While LLM responses will need to be reviewed and edited by clinicians for accuracy, the quality of their output will increase over time with greater exposure to training data. It is imperative that LLMs are trained only on high-quality data and strictly vetted to prevent performance drift or perpetuation of biases over time. The impact of these models on human critical thinking and the many ethical, legal, and societal implications are the subject of much debate^33^.

**Case Vignette Part 4:** Mr. A.I. is a former college lacrosse player and is interested in how lifestyle changes can decrease the burden of medications he takes. He has a smartwatch and wants to learn more about how this may help him prevent progression of his heart disease.

## Digital health innovation

As technology becomes increasingly engrained in daily life, DHI has the potential to empower patients by giving them more access, knowledge, and control over their health data, allowing them to become greater participants in their own health. DHI also offers a new understanding of physiology and behavior patterns in life outside the clinical setting, a previously inaccessible frontier to clinicians^34^.

Multiple studies support the use of digital health technology to improve cardiovascular outcomes. For example, the MiCORE (Myocardial infarction, COmbined- device, Recovery Enhancement) study integrated a smartphone application, smartwatch, and blood pressure monitor to support guideline-directed care during hospitalization for acute myocardial infarction and through 30-days post-discharge via (1) medication reminders, (2) vital sign and activity tracking, (3) education, and (4) outpatient care coordination. This DHI was associated with fewer all-cause 30-day readmissions than the control group (6.5 versus 16.8%, respectively)^23^ and an average net cost savings of $7,319 per patient compared to the standard of care alone^35^. In fact, approximately 50% of US adults with a history of ASCVD adhere to optimal prevention strategies, including aspirin, statin, and antihypertensive use^36^. This underscores a significant opportunity for improvement, and remote monitoring with the support of DHI may improve therapeutic adherence and intensification.

Just as the information age has democratized education by enabling individuals of all backgrounds to access the same resources, digital health has the potential to promote health equity^37^. In this early stage of DHI, a digital divide exists between those with access to technology and those without, such as older individuals, those from lower socioeconomic backgrounds, and those with lower educational achievement^38^. AI may play a role in bridging the digital divide by expanding the variety of available digital health tools and making them more accessible by tailoring tools to individuals. For a patient-facing device, a clinically trained longitudinal AI companion could remind a patient with cardiovascular disease when to take medications and flag potentially dangerous vital sign abnormalities or weight gain to a clinician who could intervene early, preventing adverse events^39^.

**Case Vignette Part 5:** Unfortunately, 6 months later, Mr. A.I. develops substernal chest pain radiating to the left shoulder with exercise and is relieved by rest. His symptoms are thought to be stable angina. He is referred for an outpatient coronary CT angiogram to rule out left main or multi-vessel coronary artery disease and an echocardiogram to rule out left ventricular dysfunction. Can AI be used to aid diagnostic care with ECGs, coronary CT angiography, or echocardiography?

## Diagnostic workup (e.g., ECG, echocardiography, and coronary CT angiography)

**Fig. 4:**
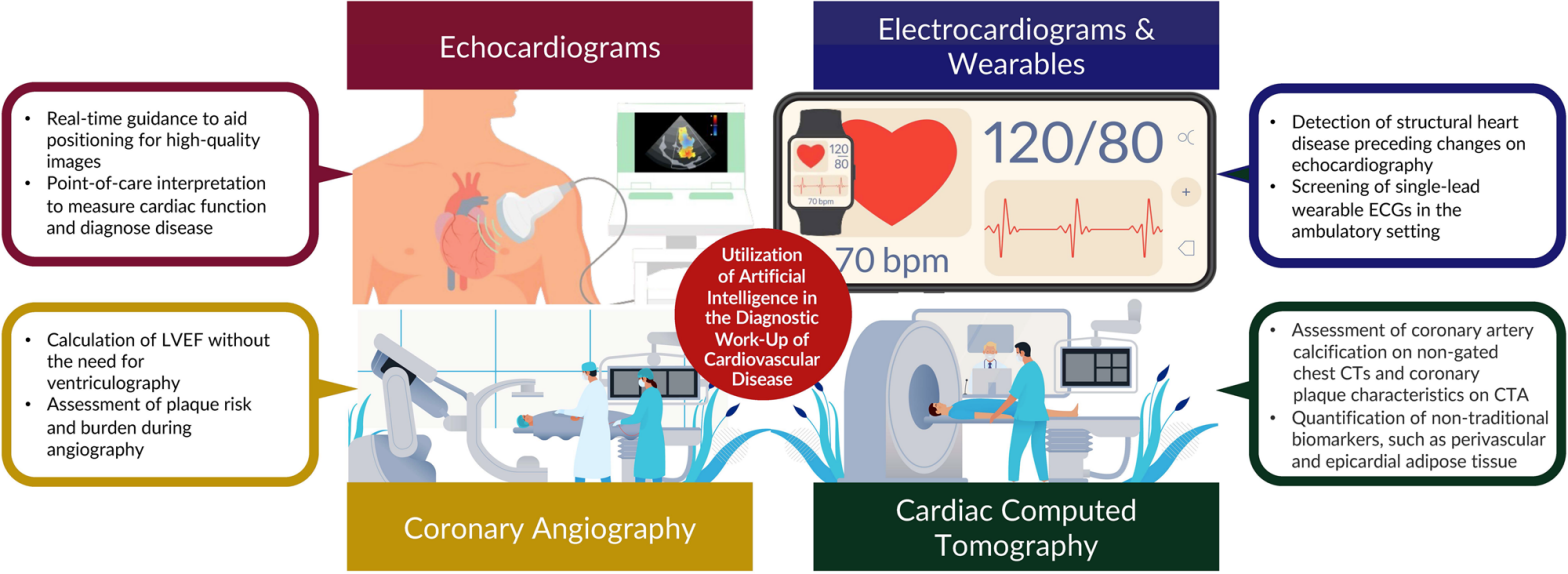
The utilization of digital health and artificial intelligence in the diagnostic work-up of cardiovascular disease.

By retaining the spatial and temporal relationships between individual data points over multiple layers of artificial “neurons”, AI models can recognize increasingly complex patterns within raw data, and ultimately provide a useful clinical prediction. For example, ECG-based AI models^40^ can be trained to recognize specific patterns within the underlying voltage waveforms, e.g., the QRS complex, or ST-segment, or uncover new patterns associated with clinical outcomes such as myocardial infarction^41^, aortic stenosis^42^, or hyperkalemia^43^, or portending high risk for some subsequent clinical outcome, e.g., development of atrial fibrillation^42^, death^44^, or ventricular arrhythmias^45^. AI can also be incorporated into echocardiographic software systems to enable untrained personnel to acquire high-quality images by providing explicit directions on the correct transducer positioning^46^. Beyond this, AI may enhance the interpretation of medical images^47–49^ and assist in the automated diagnosis of a left ventricular systolic dysfunction, various cardiomyopathies such as hypertrophic cardiomyopathy, transthyretin amyloid cardiomyopathy, and valvular heart disease^18^. The technology may aid physicians by flagging the presence of distinct clinical phenotypes based on echocardiographic patterns. For coronary computed tomography angiography (CCTA), AI has been demonstrated to enhance the quantification of soft (non-calcified) plaque burden^50^ and provide insight into the biologic and inflammatory activity of such plaque^51^ expanding the diagnostic yield of clinical reports. A secondary analysis of the CREDENCE (Computed tomogRaphic Evaluation of Atherosclerotic DEtermiNants of Myocardial IsChEmia) trial^52^ compared AI-assisted CCTA analyses to core lab-interpreted CCTA, and core lab-interpreted invasive coronary angiography with fractional flow reserve (FFR). The AI-assisted CCTA analyses enabled rapid (10.3 ± 2.7 min) and accurate identification and exclusion of high-grade stenosis when compared to blinded, core lab–interpreted invasive coronary angiography: sensitivity, specificity, PPV, negative predictive value, and accuracy of 94, 82, 69, 97, and 86%, respectively, for detection of ≥70% stenosis^52^. Beyond plaque analysis, a subsequent study (*N* = 750 patients across five sites) reported^53^ that an AI-enhanced CCTA approach compared to conventional CCTA evaluation increased physicians’ diagnostic confidence two-to five-fold, decreased the need for downstream non-invasive and invasive testing (reduced by 37.1%, *p* < 0.001) and increased the prescription of preventive therapies such as aspirin (increased by 23.0%, *p* < 0.001) and statins (increased by 28.1%, *p* < 0.001). Further studies demonstrate the utility of AI-assisted atherosclerotic plaque analysis for predicting MACE^54^ and culprit lesions for acute coronary syndromes^55^. Similarly, imaging-based AI models have also been developed for other imaging modalities, such as cardiac magnetic resonance and nuclear cardiology modalities, based on patterns of pixel intensity within either still frame images or imaging videos (Fig. 4). Lastly, AI models thereby have the power to provide automated, standardized, and rapid clinical assessments that expand the use of raw physiologic waveform and medical imaging data beyond that of historical “rules-based” automated medical diagnostic algorithms.

## Ensuring the safe and widespread implementation of digital health innovation and artificial intelligence

**Fig. 5:**
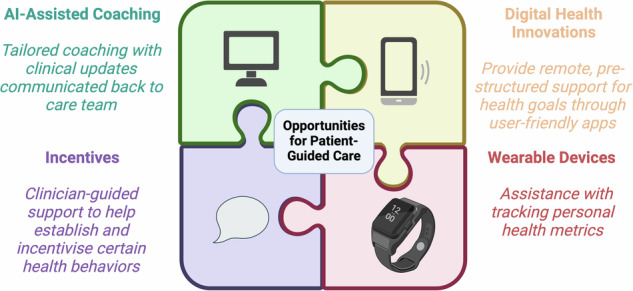
Multi-faceted use of technology, DHI and AI to promote patient-focused cardiovascular care.

Technological advances in DHI and AI will continue to integrate into everyday clinical care (Fig. 5). While these advances have the potential to improve efficiency, accuracy, and patient satisfaction, some challenges exist when ensuring their safe and widespread implementation.

To explore the variance of normal versus pathologic patterns within raw data, the development of novel AI models requires large datasets with thousands or even millions of patients in whom the clinical diagnosis or outcome is known (i.e., labeled data). Extracting raw data from clinical repositories for use in AI model vetting can be a costly process requiring expert information technology support. Even when such large datasets are available, the number of trainable parameters in most AI models may exceed the training cohort size by orders of magnitude. Special care must be taken during model development to prevent the overfitting of model weights to an individual dataset. Testing and validation of new AI models is paramount, particularly in diverse patient populations^56,57^, in order to ensure reproducibility. Obtaining parallel datasets for AI model validation often requires collaboration and data sharing between multiple centers, which may be restricted by hospital policies or laws governing patient-protected health information. Interest in the commercialization of AI models has also led many groups to withhold the publication of trained models, further impeding collaborative efforts. Another way this may be achieved is by prospectively planning performance checks over time in the sites where a model will be deployed. While this may not be necessary for all algorithms, such as for AI calculation of a CAC score which is relatively conserved across populations and over time, it may be more important for applications such as risk scores and language models that may change as populations evolve and new data becomes available.

Skepticism persists among patients and clinicians for multifactorial reasons—for example, concerns regarding competing interests in collaborations between healthcare and industry, limited interpretability of AI, and concerns regarding model performance—which can be mitigated in several ways^58^. The use of any DHI/AI technologies should be declared to both patients and clinicians participating in their use as the standard of care. For instance, once incidental CAC is reported with the aid of AI, a declaration statement should be posted at the end of each radiology report stating, “this report was created with the aid of a proprietary AI algorithm that is validated and approved by the FDA and overseen by board-certified radiologists”. Agencies governing US healthcare should revise laws and regulations regarding data science and medical education curricula to incorporate further learning on the role of DHI/AI in medicine. To promote equity, diverse stakeholders (e.g., women in cardiology working groups through the American Heart Association or American College of Cardiology, and the Association of Black Cardiologists) should be incorporated in developing digital health systems and targeted outreach to underrepresented groups encouraged. Several administrative hurdles and legal obstacles regarding the use of AI in routine clinical pathways hinder successful implementation, such as who assumes responsibility if an algorithm makes an error^59^. Some possible solutions include creating standardized protocols for data sharing, accessing anonymized data and validation, and ensuring that personal health records are encrypted. Furthermore, consideration should be given to involving more physician stakeholders with a background in DHI/AI in the regulatory bodies of each system (e.g., Federal Trade Commission, FDA, local institutional review boards, and advocacy groups) and promoting changes in the reimbursement structure and policies needed to reimburse services rendered by AI assistance.

## Emerging areas for digital health innovation and artificial intelligence

AI will eventually penetrate every aspect of cardiovascular care. While cardiovascular care throughout the vignette may be achieved without DHI/AI, the incorporation of these technologies is an additive tool that may improve patients’ experiences and clinical efficiency, and reduce the burden on clinicians. In addition to the uses in the aforementioned case vignette, there are emerging areas, such as multimodal AI, which can synthesize information from multiple structured and unstructured sources (for example, not just interpreting an ECG from a specific machine but also from different formats, such as a photocopy or PDF, and synthesizing this with other clinical data), that may have meaningful future applications in clinical cardiology. Medical teams of the future may be multidisciplinary groups of human clinicians and intelligent machines working in collaboration to make the best decision for a patient. As clinicians, it is our responsibility to ensure that AI improves outcomes for our patients while protecting them from potential harms, such as performance shifts, overfitting, and bias. Likewise, by becoming involved now, we can help ensure that AI improves cardiology for clinicians by taking over repetitive tasks to allow clinicians to focus on the humanistic aspects of patient care.

## Conclusion

Multiple worldwide organizations, including the WHO, have recognized the growing need for the implementation of technology, DHI, and AI to aid preventive cardiovascular care and management. This case-based approach demonstrates the utility of various technological advances throughout several phases of a patient’s cardiovascular care. In the near future, DHI and AI may be used throughout an individual’s interface with healthcare to minimize the burden on both patients and caregivers, reduce structural inequities in access to care, and improve the efficiency of evidence-based care delivery. Optimally, these tools have the potential to be used synergistically to improve quality, promote health equity, and provide enhanced preventive cardiovascular care.

## Data Availability

No datasets were generated or analysed during the current study.
